# Molecular characterization of midgut microbiota of *Aedes albopictus* and *Aedes aegypti* from Arunachal Pradesh, India

**DOI:** 10.1186/s13071-015-1252-0

**Published:** 2015-12-18

**Authors:** Kamlesh K. Yadav, Ajitabh Bora, Sibnarayan Datta, Kshitij Chandel, Hemant K. Gogoi, G. B. K. S. Prasad, Vijay Veer

**Affiliations:** Biotechnology Division, Defence Research Laboratory, DRDO, Post Bag 2, Tezpur, Assam India; Molecular Virology Laboratory, Defence Research Laboratory, DRDO, Post Bag 2, Tezpur, Assam India; Vector Management Division, Defence Research and Development Establishment, Jhansi Road, Gwalior, M.P. India; School of Studies in Biochemistry, Jiwaji University, Gwalior, M.P. India

**Keywords:** *Aedes*, Parasites, 16S rRNA, Midgut microbiota

## Abstract

**Background:**

Microbiota inhabiting midguts of mosquitoes play a key role in the host - parasite interaction and enhance vectorial capacity of viral diseases like dengue and chikungunya fevers. Mosquito midgut is considered to be an important site for host-pathogen interaction and pathogen survival is thought to be an outcome of this interaction. In the present study we examined the bacterial community in the midgut of *Aedes* mosquitoes in Arunanchal Pradesh, India, a subtropical zone where dengue fever is reported to be emerging.

**Method:**

Larvae and pupa of *Aedes* mosquitoes were collected from a biodiversity hotspot, Bhalukpong, Arunachal Pradesh, India. 16S rRNA gene sequences were used for identification of isolated bacterial population from each species of mosquitoes. We used various diversity indices to assess the diversity and richness of the bacterial isolates in both mosquito species.

**Result:**

On the basis of 16S rRNA gene sequence analysis a total of 24 bacterial species from 13 genera were identified belonging to 10 families of four major phyla. Phylum Proteobacteria was dominant followed by Firmicutes, Bacteroidetes and Actinobacteria. The midgut bacteria belonging to the phylum Proteobacteria and Firmicutes were isolated from both *Ae. albopictus* and *Ae. aegypti*, whereas, bacteria belonging to phylum Bacteroidetes and Actinobacteria were isolated only from *Ae. albopictus* and *Ae. aegypti* respectively. *Enterobacter cloacae* was the dominant bacterial species in both *Ae. albopictus* (33.65 %) and *Ae. aegypti* (56.45 %). *Bacillus aryabhattai* (22.78 %) was the second most common bacterial species in *Ae. albopictus* whereas, in *Ae. aegypti* the second most common bacterial species was *Stenotrophomonas maltophilia* (7.44 %).

**Conclusion:**

The family Enterobacteriaceae of phylum Proteobacteria was dominant in both species of *Aedes* mosquitoes. To the best of our knowledge, this is the first attempt to study midgut microbiota from a biodiversity hotspot in Northeastern India. Some bacterial genera *Enterobacter* and *Acinetobacter* isolated in this study are known to play important roles in parasite-vector interaction. Information on midgut microflora may lead towards the development of novel, safe, and effective strategies to manipulate the vectorial capacity of mosquitoes.

## Background

The mosquitoes *Aedes aegypti* and *Aedes albopictus* are considered major public health problems. Recent reports have provided evidence of the involvement of *Ae. aegypti* and *Ae. albopictus* in outbreaks of arboviral diseases in different parts of the globe [1, 2] including dengue and chikungunya fevers. Population growth, rapid urbanization, human travel and failures of preventive public-health measures are the major factors for increasing dengue fever cases [3–8]. Of note, dengue cases are increasing not only in urban areas, but also in rural areas [9]. The burden of dengue fever has increased drastically in the last few decades, and about 40 % population living in more than 100 countries is affected. The highest prevalences are documented in South-East Asia, America and regions of the Western Pacific. Currently, about 2.5 billion people are estimated to be at risk of dengue infection with 50–100 million infections occurring annually, worldwide [10].

Dengue fever was first documented in India in the year 1945 [11]. Subsequently, during 1963–64, dengue fever was reported from the Eastern coast of India [9]. According to a survey report in 1963, dengue activity in North East India was recorded in Assam (Darrang district) and Arunachal Pradesh (Lohit district) [12, 13]. Currently an increasing number of Dengue cases are being reported from other parts of North Eastern India [13–17].

From the literature available, it has become evident that midgut bacteria of disease vectors directly and/or indirectly affects host-pathogen interactions, and ultimately vector competency, thereby significantly influencing disease transmission potential [18–22]. The mosquito is thought to modulate the composition of its midgut bacterial population [23]. In the highly specialized gut structure of insects, pH, presence of digestive enzymes and food ingested by the host, are factors shown to significantly influence the diversity of microbial communities of insects [24]. The involvement of midgut bacteria in various important functions in relation to host and parasite interaction has been reported, and further studies on midgut microbial composition, its acquisition, and ability to modulate host parasite interaction have become a focus of research for several laboratories, worldwide [25–29].

Considering the global surge in incidences of emerging and re-emerging vector-borne diseases [30], researchers have examined the microbial diversity in different insect vectors, especially mosquitoes, to understand the host-microbe-pathogen interactions as well as to investigate the potential application of the host microbes in vector management [19]. Midgut microbiota affects the vectorial capacity of *Anopheles* mosquitoes by hampering the development of malaria parasites [26]. Inhibition of parasite growth and development has been suggested to be achieved by inducing the production of an effector molecule by genetically modified midgut bacteria [31].

In India, attempts to scrutinize the midgut microflora has remained mainly focused on *Culex* and *Anopheles* mosquitoes, which act as vectors for Japanese encephalitis virus, filariasis nematodes and malaria protozoa [25, 27, 32–34]. Despite being the major vector for dengue, midgut microbial diversity studies in different species of *Aedes* mosquitoes are rare, especially from India. Although, a recent study reported the midgut microbial diversity in different *Ae. aegypti* strains (MOYO, MOYO-R, and MOYO-S) with varying vector competency [29], to the best of our knowledge, similar studies on field collected *Ae. aegypti* and *Ae. albopictus* have not been reported from India.

Therefore, we undertook this comprehensive study to understand and compare the microflora associated with midgut of field collected *Ae. aegypti* and *Ae. albopictus*. We collected larvae samples of *Ae. aegypti* and *Ae. albopictus* from the same habitats to study the bacterial diversity in the midgut of these two mosquito species. We used 16S rRNA gene sequence based techniques and various diversity indices to explore the species richness, dominance and evenness of bacterial species in the midgut. We report finding differential predominance of bacterial strains in the two species of *Aedes*, which might have important implications in vector management strategies.

## Methods

### Sample collection and midgut dissection

The fourth instar larvae and pupa of *Aedes* mosquitoes were collected from 10 different breeding spots (dump tyres and water storing pots) during the post monsoon season from Bhalukpong, West Kameng district of Arunachal Pradesh, India (Latitude: 27.01° N, Longitude: 92.65° E), a small town located along the southern reaches of the Himalayas. The collected samples were brought to the laboratory and emerged pupae were transferred to the pre-sterilized net cage for adult emergence. The emerged adult mosquitoes were anesthetized using chloroform and the species were identified morpho-taxonomically. Adults emerged from nine samples were either *Ae. aegypti* or *Ae. albopictus*, only one sample had mixed population of *Ae. aegypti* and *Ae. albopictus* mosquitoes. Adults from mixed population were segregated and analyzed for midgut bacterial diversity. For isolation of midgut bacterial population, a total of 30 adult female mosquitoes were dissected from each species of mosquitoes. Prior to dissection, all the dissecting apparatus, plasticwares, glasswares, buffers and solutions, were sterilized by autoclaving and UV treatment. All the 60 mosquito samples were surface sterilized with 75 % ethanol for 5 min followed by washing with phosphate buffered saline (PBS) twice prior to dissection. Individually dissected midguts were transferred to 1.5 ml micro-centrifuge tube containing 100 μl of PBS and homogenized with a sterilized micropestle [27].

### Isolation of midgut bacteria

Gut homogenates were serially diluted (10 folds) in PBS, were directly pour plated on sterile nutrient agar media (Himedia, India), and incubated at 37 °C for 24–48 h. All procedures were done in a sterile environment, strictly following aseptic practices and negative controls (PBS) were included throughout the experiment. Bacterial colonies obtained on the plate were differentiated according to their colony morphology like shape, size, colour, margin, opacity, elevation etc. Morphologically distinct colonies were selected for repeated subculture on nutrient agar plates until a presumably pure colony was obtained. Finally, a total of 82 representative colonies were selected for sequencing based on morphological characteristics.

### Genomic DNA isolation and PCR amplification of 16S rRNA

Genomic DNA was isolated from bacterial pellets obtained from the centrifugation of fresh over night culture in nutrient broth and re-suspended in Tris-EDTA (TE buffer, pH-8). For the lysis of bacterial cells, a repeated heat shock (freezing and thawing) method was used followed by lysozyme and proteinase-K treatment. Genomic DNA was precipitated in chilled isopropanol and the DNA pellet was air dried and suspended in TE buffer [35]. The small subunit of 16S rRNA gene segment was amplified using primer set 16S1 (5′-GAGTTTGATCCTGGCTCA-3′) and 16S2 (5′-CGGCTACCTTGTTACGACTT-3′) in 50 μl PCR reaction mixture [36]. The program for PCR reaction was set as, initial denaturation at 94 °C for 2.5 min, followed by 35 cycles of 94 °C for 30 s, 55 °C for 30 s, 72 °C for 1 min, followed by a final extension step at 72 °C for 7 min. The quality and quantity of amplified PCR product were checked on 1.2 % agarose gel electrophoresis and visualized through staining with Ethidium bromide (final concentration 0.5 μg/ml). PCR products were gel purified, cycle sequencing was done using BigDye^®^ terminator kit following manufacturer’s instructions (Applied Biosystems Inc. ABI, Foster City, CA) and products analyzed on an ABI 3500xL Genetic Analyzer platform (at Chromus Biotech Pvt Ltd, Bangalore, India). Amplicons were sequenced from both directions using forward and reverse primers.

### Sequence analyses

Sequence data generated in present study were submitted to the GenBank under the accession numbers (KP717387-KP717416). Homologous sequences were searched in the GenBank database using BLAST (http://www.ncbi.nlm.nih.gov/BLAST) and also using the EzTaxon server (http://www.ezbiocloud.net/eztaxon) [37]. Bacterial identification was done on the basis of more than 99 % similarity with sequences submitted in the GenBank (Table 1).

**Table 1:** Comparison of occurrence of different bacterial species in *Ae. albopictus* and *Ae. aegypti*

Phylum	Closest related bacterial species^a^	% of occurrence in *Ae. albopictus* (95 % CI^b^)	% of occurrence in *Ae. aegypti* (95 % CI^b^)	Comparative estimate of occurrence in *Ae. albopictus & Ae. aegypti* Odds ratio (95 % CI)
Proteobacteria	*Enterobacter cloacae*	33.65 (0.311–0.363)	56.44 (0.536–0.592)	2.555 (2.170–3.008)
	*Enterobacter hormaechei*	2.00 (0.014–0.029)	ND	ND
	*Enterobacter asburiae*	1.04 (0.006–0.018)	ND	ND
	*Enterobacter xiangfangensis*	ND	6.12 (0.049–0.076	ND
	*Klebsiella oxytoca*	2.80 (0.020–0.039)	ND	ND
	*Klebsiella michiganensis*	4.16 (0.032–0.054)	0.99 (0.006–0.017)	0.231 (0.123–0.435)
	*Klebsiella pneumoniae*	ND	0.58 (0.003–0.012)	ND
	*Pantoea dispersa*	ND	2.40 (0.017–0.034)	ND
	*Pseudomonas aeruginosa*	2.16 (0.015–0.031)	ND	ND
	*Pseudomonas monteilii*	4.56 (0.035–0.059)	6.03 (0.048–0.075)	1.345 (0.942–1.920)
	*Pseudomonas geniculata*	3.68 (0.028–0.049)	ND	ND
	*Pseudomonas mosselii*	ND	0.91 (0.005–0.016)	ND
	*Acinetobacter pittii*	5.84 (0.047–0.073)	ND	ND
	*Aeromonas veronii*	ND	0.99 (0.006–0.017)	ND
	*Stenotrophomonas maltophilia*	ND	7.44 (0.061–0.091)	ND
	*Alcaligenes faecalis*	1.36 (0.009–0.022)	ND	ND
Firmicutes	*Bacillus aryabhattai*	22.78 (0.205–0.252)	0.91 (0.005–0.016)	0.031 (0.017–0.057)
	*Bacillus cereus*	0.72 (0.004–0.014)	ND	ND
	*Bacillus tequilensis*	0.80 (0.004–0.015)	ND	ND
	*Bacillus aerophilus*	ND	4.63 (0.036–0.060)	ND
	*Lysinibacillus fusiformis*	1.52 (0.010–0.024)	6.03 (0.048–0.075)	4.163 (2.497–6.941)
	*Staphylococcus hominis*	9.35 (0.079–0.111)	5.12 (0.040–0.065)	0.523 (0.381–0.720)
Bacteroidetes	*Elizabethkingia anophelis*	3.60 (0.027–0.048)	ND	ND
Actinobacteria	*Micrococcus yunnanensis*	ND	1.40 (0.009–0.022)	ND

^a^all bacterial species were identified on the basis of a % identity higher than 99 %

^**b**^ *CI* Confidence interval

### Statistical analysis

Mann–Whitney rank sum tests was performed to estimate the differences in prevalence of bacterial species between *Ae. albopictus* and *Ae. aegypti* midguts (p < 0.05, 95 % confidence interval). To calculate confidence intervals for comparison of the presence of each bacterial species in the two groups of *Aedes* mosquito, an Excel spreadsheet tool was used [38]. Various diversity indices *i.e.* Simpson Index [39], Shannon Index, Sørensen Index, and Evenness [40] of bacterial communities from *Ae. albopiictus* and *Ae. aegypti* midgut were calculated [27]. The following formula was used to calculate Good’s coverage: percentage of coverage (1-n/N)* 100, where n represents a single bacterial isolate and N denotes total bacterial isolates from one mosquito species [41].

## Results

Bacterial isolates obtained from the midguts of *Ae. albopictus* and *Ae. aegypti* were screened based on their colony characteristics and identified on the basis of 16S rRNA gene sequences. Based on 16S rRNA gene sequence analysis, a total of 24 species from 13 genera were identified, belonging to 4 major phyla: Proteobacteria, Firmicutes, Bacteroidetes and Actinobacteria (Tables 1). Proteobacteria (66.7 %) was the dominant bacterial phylum followed by Firmicutes (25 %). Actinobacteria and Bacteroidetes were the least represented phylum as only one species was identified from each of them.

### Bacterial isolates from midgut of *Ae. albopictus*

A total 16 different bacterial species were identified from the midgut of *Ae. albopictus* out of which phylum Proteobacteria (62.5 %) was the most prominent and 10 of 16 bacterial species belonged to this phylum. The second largest phylum was Firmicutes (31.25 %) containing 5 bacterial species of total 16 identified bacterial species. The least number of bacteria were from the phylum Bacteroidetes (6.25 %). In Proteobacteria phylum, bacteria belonging to the class Gamma Proteobacteria was dominant (56.25 %) followed by Beta Proteobacteria (6.25 %). When the total identified bacterial species were classified according to their family, Enterobacteriaceae (31.25 %) with 5 species was found to be the most abundant, followed by Bacillaceae (25.00 %) with 4 species, Pseudomonadaceae (18.75 %) with 3 species, Staphylococcaceae (6.25 %), Moraxellaceae (6.25 %), Alcaligenaceae (6.25 %) and Flavobacteriaceae (6.25 %) with single number of bacterial species. *Enterobacter cloacae* was found to be the most dominant bacterial species followed by *Bacillus aryabhattai.*

### Bacterial isolates from midgut of *Ae. aegypti*

A total 14 different bacterial species were identified from the midgut of *Ae. aegypti* out of which 9 bacterial species from the phylum Proteobacteria (64.29 %) and 4 different bacterial species from the phylum Firmicutes (28.57 %). Proteobacteria was the largest phyla observed in *Ae. aegypti* and, as in *Ae. albopictus,* Firmicutes was the second. The least represented phylum overall was Actinobacteria (7.14 %) represented by a single bacterial species. All the bacterial species belonging to the phylum Proteobacteria were found to be of class Gamma Proteobacteria*.* When classified according to family, the maximum number of bacterial species in *Ae. aegypti*, belonged to family Enterobacteriaceae (35.71 %) followed by the family Bacillaceae (21.43 %), Pseudomonadaceae (14.29 %), Aeromonadaceae (7.14 %), Xanthomonadaceae (7.14 %), Staphylococcaceae (7.14 %), and Micrococcaceae (7.14 %). Among the bacterial isolates from *Ae aegypti*, *Enterobacter cloacae* was the most frequently isolated bacterial species followed by *Stenotrophomonas maltophilia.*

### Statistical analysis

Bacterial prevalence of both the mosquitoes species were compared using Mann Whitney rand sum test. We did not observed statistically significant difference in bacterial species prevalence between *Ae. albopictus* and *Ae. aegypti* mosquitoes (p = 0.56). Various diversity indices were calculated for the estimation of diversity among the bacterial communities in the midguts of *Ae. albopictus* and *Ae. aegypti* (Table 2). The Good’s coverage was found to be 79.06 % and 82.05 % for *Ae. albopictus* and *Ae. aegypti,* respectively. Although not significant, but Simpson, Shannon, Margalef diversity indices were slightly better for *Ae. albopictus,* compared to *Ae. aegypti.* The species dominance and evenness values were greater in *Ae. aegypti*, compared to *Ae. albopictus* (Table 2).

**Table 2:** Diversity indices, total taxa and Good’s coverage of midgut bacterial isolates of *Ae. albopictus* and *Ae. aegypti*

	*Aedes* mosquitoes	
	*Ae. albopictus*	*Ae. aegypti*
Total taxa identified	16	14
Individuals	43	39
Total number of bacteria isolated (N)	43	39
Bacterial species represented by single isolate (n)	9	7
Good’s coverage [(1-n/N)*100]	79.06	82.05
Dominance	0.1466	0.1729
Simpson diversity index	0.8534	0.8271
Shannon diversity index	2.306	2.185
Evenness	0.6268	0.6352
Margalef diversity index	3.988	3.548

## Discussion

It has been reported that the midgut bacteria of mosquitoes play a significant role in the vector-parasite interaction [26, 42]. The present work was carried out to study the diversity of midgut bacteria of two species of mosquito *viz. Ae. albopictus* and *Ae. aegypti* collected from the foothills of Arunanchal Pradesh, North East India. In this study, we only focused on the characterization of culture-dependent aerobic bacteria from the midgut of both species of *Aedes* mosquitoes, because only culturable bacteria can be used for further applications in management of disease transmission such as paratrangenesis.

In our observation, *Ae. albopictus* was more frequently found as compared to *Ae. aegypti* among the collection sites. The low abundance of *Ae. aegypti* may be due to the fact that this species is usually found in urban areas, unlike *Ae. albopictus* which is commonly found in rural habitats and prefer breeding in natural habitats like bamboo, stumps, tree holes, and bromeliads [43, 44]. In present study, we have analyzed the midgut of *Ae. aegypti* and *Ae. albopictus* females, sharing the same habitat during their larval development.

A total of 24 different bacterial species identified by a 16S rRNA gene sequence analysis was obtained from both species of *Aedes* mosquito and most of the bacterial genera had already been reported from the midgut of *Aedes* as well as other mosquito species. The bacterial genera of *Enterobacter, Bacillus, Pseudomonas, Staphylococcus, Klebsiella, Pantoea, Acinetobacter* and *Aeromonas* have been reported from midgut of mosquitoes and the results of the present study corroborate with findings reported by other workers [25, 27, 34, 45–57]. Some bacteria species are closely associated with mosquito gut environment and common inhabitants of *Aedes* as well as other mosquito species [27]. From the results, we observed that in both mosquitoes species, maximum bacterial species belong to families Enterobacteriaceae and Bacillaceae. It has been reported that, in the mosquito’s midgut, the bacteria are primarily acquired either through vertical inheritance or through acquisition from the environment [58]. The bacterial species *Enterobacter cloacae, Klebsiella michiganensis, Pseudomonas monteilii, Bacillus aryabhattai, Lysinibacillus fusiformis, Staphylococcus hominis* have been isolated from both *Ae. albopictus* and *Ae. aegypti*. Whereas, there were some other species, which were retrieved either from *Ae. albopictus* or *Ae. aegypti* mosquito gut, but their prevalence were very low. For instance, *Enterobacter hormaechei, Enterobacter asburiae, Klebsiella oxytoca, Pseudomonas aeruginosa, Pseudomonas geniculata, Acinetobacter pittii, Alcaligenes faecalis, Bacillus cereus, Bacillus tequilensis* and *Elizabethkingia anophelis* were only present in the *Ae. albopictus* whereas, *Enterobacter xiangfangensis, Klebsiella pneumonia, Pantoea dispersa, Pseudomonas mosselii, Aeromonas veronii, Stenotrophomonas maltophilia, Bacillus aerophilus* and *Micrococcus yunnanensis* were exclusively isolated from the *Ae. aegypti.*

We have observed presence of *Enterobacter xiangfangensis* from *Ae. aegypti* midgut for the first time. Earlier, this bacterial species was isolated and identified from Chinese traditional sourdough [59]. *Bacillus aryabhattai* was also not isolated from *Aedes* mosquitoes. Earlier, it was reported from *Culex quinquefasciatus* mosquito and *Capsodes infuscatus* herbivorous bug [27, 60]. Similarly, *Aeromonas veronii* was previously isolated from the midgut of *Cx. quinquefasciatus* [27] and larvae of *An. gambiae* [61] but was not isolated from midgut of *Ae. aegypti* or *Ae. albopictus. Alcaligenes faecalis,* was identified from the midgut of the sandfly, *Phlebotomus papatasi* and hindgut wall of *Dermolepida albohirtum* larvae [62, 63], previously but not recorded from mosquito gut. Similarly, *Bacillus tequilensis, Bacillus aerophilus* was previously isolated from a herbivorous bug *Capsodes infuscatus,* but was not reported from the midgut of any mosquitoes [60].

In our study, we found that *Enterobacter cloacae* was the dominant species in both *Ae. albopictus* (33.65 %) and *Ae. aegypti* (56.45 %). This finding is important since a number of studies have been done and this species of bacteria has been found to block the development of *Plasmodium falciparum* in *Anopheles gambiae* and sporogonic development of *Plasmodium vivax* in *An. albimanus* [64, 65], induce the expression of mosquito immune components in midgut of *An. stephensi* [66]. In addition, *E. cloacae* has also been found to inhabit the midgut of the sand fly *Phlebotomus papatasi* and its potential application in paratransgenic approach to reduce the transmission of Leishmania has been suggested recently [67]. Apart from these potential applications, *E. cloacae* have also been successfully used to deliver, express, and spread foreign genes in termite colonies [68]. *E. cloacae* transformed with ice nucleation (IN) gene have also been shown to be useful for reduction of mulberry pyralid moth, *Glyphodes pyloalis* [69]. Considering these findings, direct application of *E. cloacae* for pathogen reduction, or through paratransgenic approach, appears to be a potential strategy towards effective management of vector-borne diseases.

The bacterial genera *Serratia* and *Enterobacter* produce hemolytic enzymes that might take part in the digestion of blood in hematophagous Diptera [52, 70, 71]. Other important bacterial genera *Acinetobacter* obtained from *Ae. albopictus* in our study are known to be involved in blood digestion. Minard *et al*., [28] reported that the bacterial species *Acinetobacter baumannii* and *A. johnsonii* isolated from *Ae. albopictus* may have a role in assimilation of nectar and blood digestion.

In the recent years, it is reported that some midgut inhabiting bacteria play an important role in disease transmission, host-parasites interaction and also affects the vectorial capacity of mosquitoes. The midgut serves as the first contact point between parasites and the epithelial surfaces, where significant parasite numbers are reduced [22]. The microbiota involved in the blocking of the *Plasmodium* development may be used in the modulation of vectorial capacity of mosquitoes [26]. Midgut microbiota is known to augment the immune response of the mosquito [26, 53, 64, 72–74]. Since immunocompetent mosquitoes are thought to be less likely to transmit other parasites such as malaria [75], similar strategies might also be helpful in dengue control through use of bacterial species that augment the mosquito immune system.

The midgut microbiota composition has an important role on susceptibility of chikungunya and dengue viruses. It has been demonstrated that the susceptibility of *Ae. aegypti* to chikungunya and dengue virus increases in the presence of midgut bacteria *Serratia odorifera* due to the suppression of immune response of *Ae. aegypti* [42, 76]. It has been showed that susceptibility to DENV-2 enhanced when *Ae. aegypti* were fed with the *Aeromonas* sp. and *Escherichia coli* [42, 77].

From the above discussion, it is clear that the midgut bacteria can be significantly involved in host-parasite interaction and may decrease or increase the vectorial capacity through various mechanisms including enhancement of immune response or precluding the development of parasites. Midgut microbiota may be genetically manipulated to express molecules against the parasites, which could be used as a novel strategy for vector management. The understanding of midgut microbiota and the mosquitoes could be used for the development of novel, eco-friendly and highly effective defense mechanism to reduce the vectorial capacity of mosquitoes and hence disease transmission control.

## Conclusion

To the best of our knowledge, this is the first time attempt towards a comprehensive study of the midgut microbiota of *Ae. albopictus* and *Ae. aegypti* in Arunanchal Pradesh, North East India. The involvement of midgut bacteria in the defense mechanism of the vector has been reported, but this information is still very limited. *Enterobacter* was found to be the predominant culturable gut bacteria genera in both *Ae. albopictus* and *Ae. aegypti* and previously reported data supports its involvement in *P. falciparum* development blockage and blood digestion. Other important bacterial genera such as *Acinetobacter* were also identified from *Ae. albopictus* which is known to take part in blood digestion of mosquitoes. Comprehensive knowledge about midgut bacteria may leads towards better understanding the direct or indirect involvement of microbiota in the immune response, nutrition and reproduction of mosquitoes.
